# Response to Clarifications on: Pectoralis Blocks Nomenclature and Applications of Regional Anesthesia Techniques

**DOI:** 10.5811/cpcem.1508

**Published:** 2023-11-30

**Authors:** Jonathan Brewer, Arun Nagdev

**Affiliations:** * Vanderbilt University Medical Center, Department of Emergency Medicine, Division of Ultrasound, Nashville, Tennessee; † Highland Hospital, Department of Emergency Medicine, Oakland, California

## To The Editor

We want to thank the author of the recent letter to the editor regarding our case series.

Regarding the first point, we do utilize the term “Pecs I and Pecs II” throughout our study and did cite the Sethuraman and Narayanan paper, which had the more descriptive nomenclature (interpectoral and pectoserratus blocks, respectively). Because our target audience is emergency physicians who are being introduced to these blocks, we wanted to use the classic terms that have been used by numerous anesthesiologists in online and published educational material (ie, NYSORA, Duke Anesthesiology, etc). We agree that the more descriptive nomenclature should be used in the future as they become more standard. With the goal of introducing this block to the specialty, we recognize that Pecs II does include a Pecs I block. However, since two of our cases are isolated to a Pecs I block we split the terms for simplicity. We do firmly agree that nomenclature established by experts is important and, therefore, we will stick to whatever the most agreed upon nomenclature is at the time of publication moving forward.

With regard to the second point, we do agree that careful attention must be paid to the sensory coverage of each block. While all our cases benefited from a Pecs I, or interpectoral plane (IPP) block, this could have been resultant due to some myofascial pain. We also recognize the limitations of a case series and note that this is an introductory paper on a subject that should be further studied on a larger scale. Furthermore, we do mention that a contraindication to the block will be overlying infection such as cellulitis. This is true of almost all procedures performed within the emergency department, and we agree that an injection into a deeper fascial plane should never be performed through infected tissue. In addition, the “Pecs Zero” block introduced by Tulgar et al has promise and is a very interesting concept that warrants further study. However, their case report only achieved sensory block of the lateral breast, upper outer quadrant, and axillary areas and lacked medial coverage.^3^ While this modification could be very useful when the presence of infected tissues exists, we believe that it warrants further research before it can be considered as a replacement as the initial letter suggests.

Finally, while we agree that the erector spinae plane block (ESPB) is an excellent block for many painful complaints, such as rib fractures, we do feel that even though thoracic ESPBs are commonly used by our anesthesiology colleagues, many emergency physicians are uncomfortable with the anatomy near the spine. The pectoralis region is often a simple target with clear anatomy. Also, there is some evidence that the Pecs II block is superior to this block in the postoperative setting for pain control of the breast.^4^ Our goal in writing our case series was to introduce a novel technique to emergency physicians that could work synergistically with oral and intravenous analgesics for painful, breast-related complaints.

We do want to thank the author of this letter again for their contribution and healthy discussion. We look forward to continued research and application within the realm of emergency medicine.
